# Emergence of uniform linearly-arranged micro-droplets entrapping DNA and living cells through water/water phase-separation

**DOI:** 10.1038/s41598-021-02990-w

**Published:** 2021-12-07

**Authors:** Mayu Shono, Ritsuki Ito, Fumika Fujita, Hiroki Sakuta, Kenichi Yoshikawa

**Affiliations:** 1 Faculty of Life and Medical Sciences, Doshisha University, Kyoto, 610-0394 Japan; 2 Department of Chemical Engineering and Materials Science, Doshisha University, Kyoto, 610-0321 Japan; 3 Organization for Research Initiatives and Development, Doshisha University, Kyoto, 610-0394 Japan; 4 Center for Integrative Medicine and Physics, Institute for Advanced Study, Kyoto University, Kyoto, 606-8501 Japan

**Keywords:** Biopolymers in vivo, Biomimetic synthesis, Biophysical chemistry, Self-assembly

## Abstract

Living cells maintain their lives through self-organization in an environment crowded with a rich variety of biological species. Recently, it was found that micro-droplets containing biomacromolecules, which vary widely in size, are generated accompanied by water/water phase-separation by simple mechanical mixing of an aqueous solution with binary polymers. Here, we report that cell-sized droplets of nearly the same size are generated as a linear array within a glass capillary upon the introduction of a binary polymer solution of polyethylene glycol (PEG) and dextran (DEX). Interestingly, when DNA molecules are added to the polymer solution, stable droplets entrapping DNA molecules are obtained. Similarly, living cells are entrapped spontaneously for the linearly-arranged cell-sized droplets. This simple method for generating micro-droplets entrapping DNA and also living cells is expected to stimulate further study on the self-construction of protocells and micro organoids.

## Introduction

Recent studies on the construction of cell-like structure/function have attracted growing interest using terms such as protocell, minimum cell, artificial cell, synthetic cell, etc.^1–7^. These studies may provide the opportunity to examine current hypotheses regarding the ‘origin of life’ and may provide new perspectives on the problem of ‘What is life?’. In addition, the construction of an artificial cell-like structure would be useful in the fields of genetic engineering, drug development, biomimetic chemical production, etc. Several experimental methodologies^1–8^ have been proposed to achieve the compartmentalization of a water pool with a size of several tens of μm entrapping biological materials. Water/oil (w/o) droplets have been rather frequently used to obtain aqueous cell-sized compartmentalization. It has been demonstrated that such w/o droplets can be transformed into giant vesicles covered by a phospholipid membrane by adopting suitable experimental methodologies to transport them into an aqueous environment^5,9,10^. Actually, successful experimental observations have been attained such as directional insertion of membrane channel proteins^11^ and cell-free protein production inside the compartment^12^. Several researchers have been seeking to develop a methodology for producing w/o droplets and transforming them into giant liposomes. It has been revealed that stable w/o droplets of a desired size can be generated through the application of microfluidic experimental techniques^13,14^. On the other hand, there has also been an increase in studies with water/water (w/w) droplets to construct protocells, which can avoid the contaminating effect of oil. Aggregates or colloidal assemblies of several different macromolecules are called coacervates^15^. Almost half a century ago, Oparin^15^ proposed the generation of droplets and aggregates similar to coacervates (nowadays frequently referred to as liquid–liquid phase separation, LLPS) due to a difference of the interaction parameters in the solutes and solvent, as in the usual Flory–Huggins model in polymer physics, including electrostatic interaction, hydrophobic interaction, hydrogen bond, hydration effect, etc.^16,17^. Interestingly, many cellular organelles are regarded to be formed through the process of coacervation or LLPS^18^. In other words, organelles such as P granules, stress granules and Cajal bodies are found to be stabilized without a phospholipid membrane^19,20^. Thus, it would be a promising strategy to use the phenomenon of w/w micro phase-separation for the construction of protocells^21^.

As a somewhat different research stream, aqueous two-phase systems (ATPS), or water/water (w/w) phase segregation, generated in the presence of a relatively crowded environment with a binary polymer solution have been studied in relation to the formation of protocells as well as artificial models of organelles^22–26^. Recently, studies toward the application of microfluidics to ATPS (w/w droplets) have been reported so as to mimic cellular structure and function^6^. Notably, the main mechanism of phase segregation or LLPS in a binary polymer solution is the so-called ‘depletion effect’^27,28^ of polymer molecules, which is a kind of entropic effect on the conformational freedom of polymer molecules in a relatively crowded condition. Under crowded conditions, chemically attractive interaction among the same polymer molecules has a minor effect compared to the entropic effect by the depletion effect, in contrast to the current hypothesis of coacervates. Recently, it was reported that the specific entrapment/localization of DNA and actin filaments occurs in w/w cell-sized droplets, by using an aqueous solution of binary solvable polymers^29^. It has also been shown that, in a similar polyethylene glycol (PEG)/dextran (DEX) solution, prion protein exhibits characteristic localization depending on the solution conditions^30^. In an aqueous solution with DEX and PEG, these biomolecules are specifically accumulated in micro-droplets rich with DEX. As an extension of that study, it was found that stable cell-sized droplets entrapping DNA emerge spontaneously through simple mixing of the solution^29^. It was also revealed that, under suitable solution conditions containing phospholipid, cell-sized droplets covered by phospholipid and entrapping DNA molecules are generated in a self-organized manner^31^. In these studies, although the self-emergence of cell-like structures was confirmed, the size of the w/w droplets or protocells exhibits rather wide dispersion with a range of μm-mm, because the w/w droplets were generated after simple mechanical mixing. In the present report, we show the results of our new experiment on w/w phase segregation by revealing the appearance of almost uniform-sized w/w droplets in a narrow glass capillary. Effective entrapment of DNA and living cells in these arranged, uniform-sized w/w droplets will be demonstrated.

## Results

**Figure 1:**
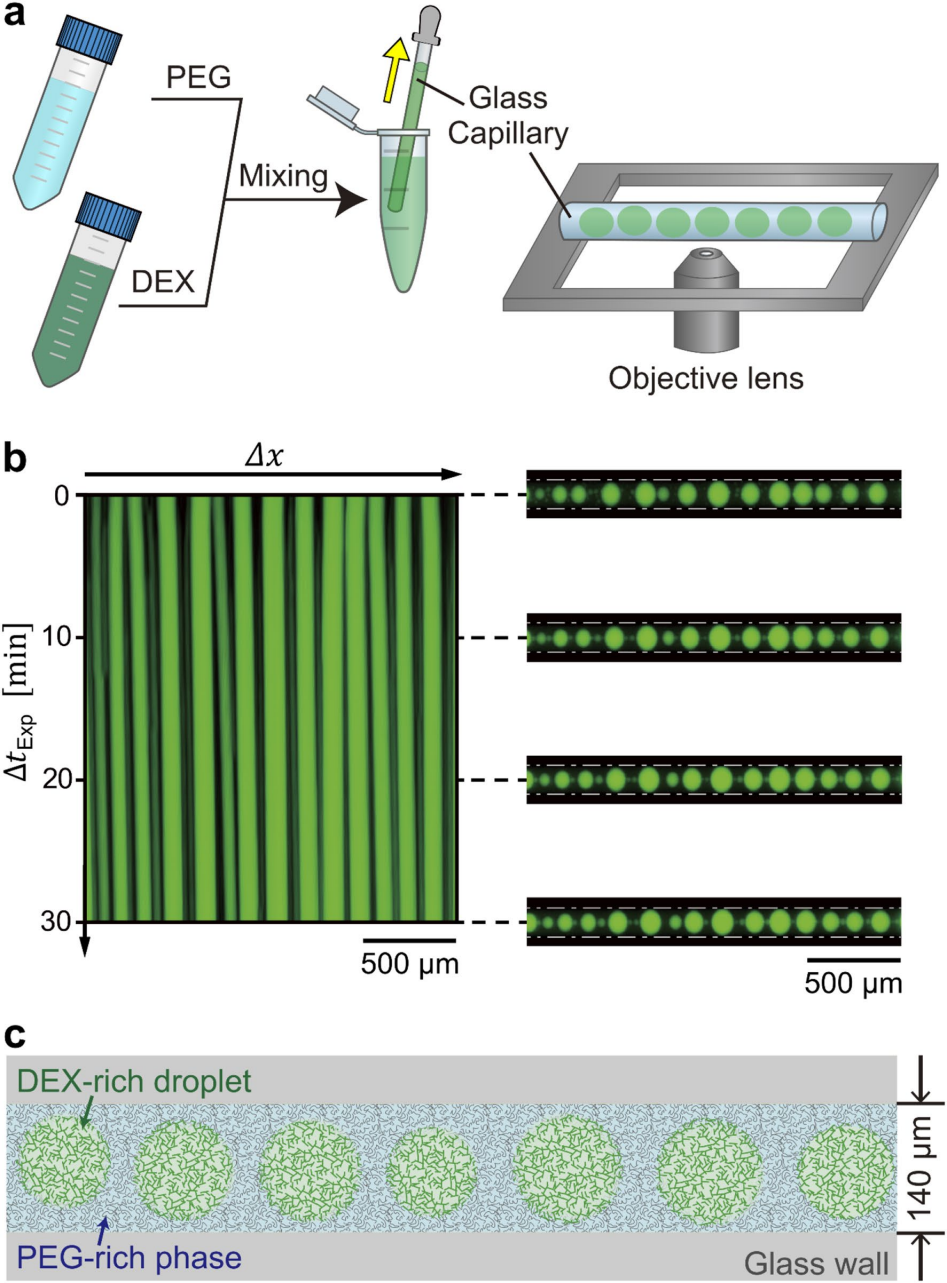
Emergence of linearly-arranged micro w/w droplets inside a glass capillary. (**a**) Schematic representation of the experimental procedure. After mechanical mixing of PEG and DEX solutions at a final composition of PEG/DEX = 5 wt%:5 wt%, the solution was sucked up into a glass capillary (inner diameter: 140 µm) and then both ends of the capillary were sealed. The glass capillary was placed horizontally on the stage of an optical microscope. (**b**) Left: Spatiotemporal diagram, Right: Snapshots of the capillary at different times. Time is given as the duration after the start of the microscopic observation, which is ca. 2 min after the timing of mechanical mixing. Dashed lines indicate the inner glass wall. DEX was labeled with fluorescein isocyanate-bound DEX (FITC-DEX, Mw = 250,000). (**c**) Schematic drawing of the linearly-arranged w/w droplets. The gray area represents the glass wall of the capillary.

Figure 1 exemplifies the formation of linearly-arranged cell-sized w/w droplets together with the experimental scheme. We adopted a binary polymer solution with PEG (Mw 7300–9300 Da) and DEX (Mw 180,000–210,000 Da) for macroscopic phase-separation under equilibrium^29,32^. The left panel in Fig. 1b shows a spatio-temporal plot of micro phase-separation after the mechanically mixed binary polymer solution was inserted into a glass capillary with an inner diameter of 140 μm. Snapshots of the actual arranged droplets are shown on the right. The spatio-temporal plot indicates that the generated droplets are arranged along the long-axis of the capillary in a stationary manner. We confirmed that this droplet array is stable even after several days. Figure 1c shows a schematic diagram of the generated micro w/w droplets which are rich in DEX, whereas the environmental solution is rich in PEG.

**Figure 2:**
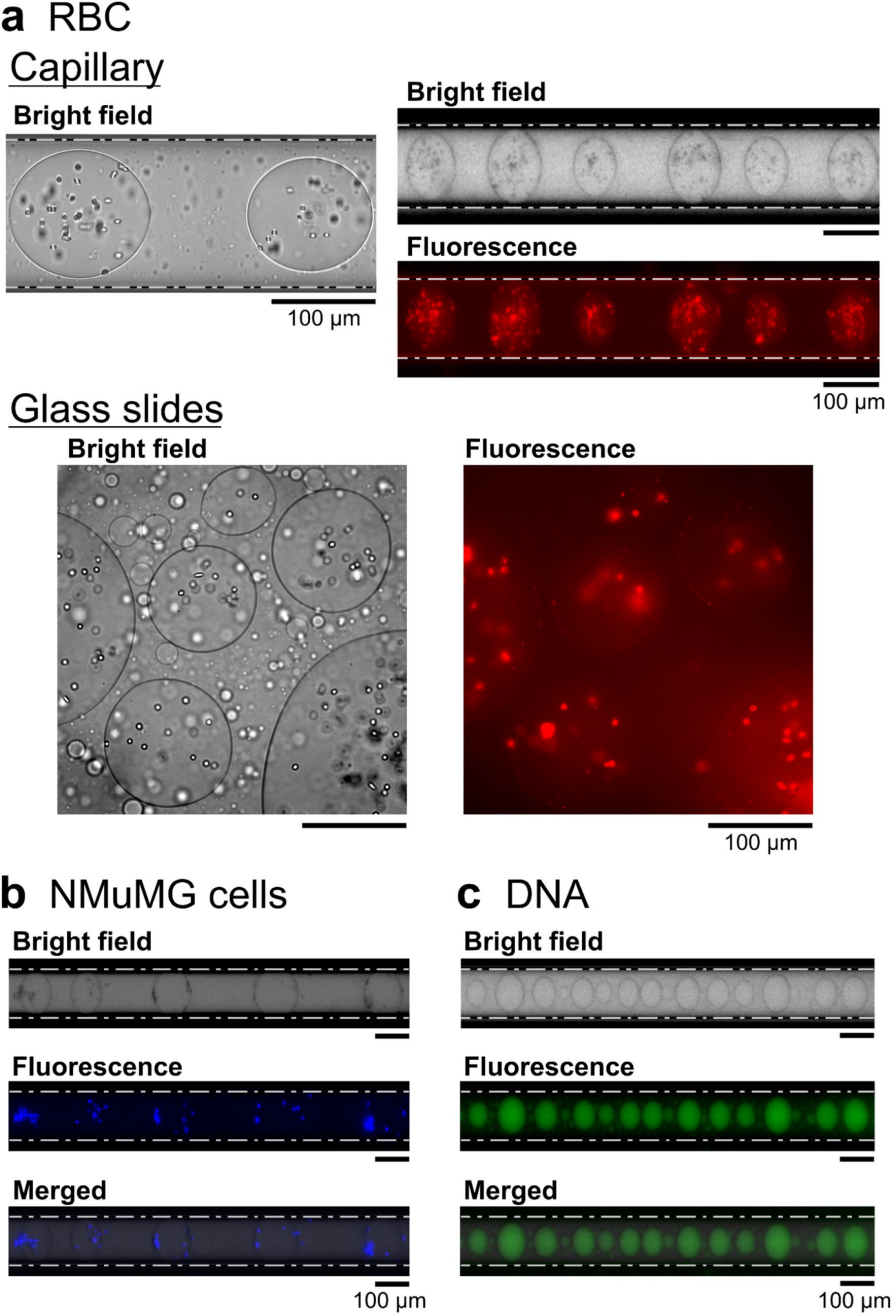
Experimental observations of the autonomous entrapment/localization of DNA and living cells. (**a**) Red blood cells (RBC) inside droplets, (**b**) epithelial cells (NMuMG) located at the interface, and (**c**) salmon sperm DNA inside the glass capillary. For comparison, the lower panel of (**a**) shows w/w droplets between planar glass plates with a depth of ca. 100 μm. The solution compositions were the same as in Fig. 1. Dashed lines indicate the inner glass wall. (**a**) Red blood cells (RBC) stained by Nile Red were distributed at both the interface and inside DEX-rich droplets. Upper panel: Using a glass capillary, uniform-sized droplets were arranged in a row. Lower panel: Various-sized droplets were formed. (**b**) and (**c**) Generation of droplets inside a glass capillary with NMuMG cells and DNA, respectively. Mouse mammary gland epithelial cells (NMuMG cells) tended to distribute at the interface of DEX-rich droplets. The NMuMG cells were fluorescence-labeled by the expression of mCherry as a fluorescent protein. Salmon sperm DNA (150 µM, 500–1000 kbp) accumulated inside the droplets of the DEX phase. DNA was labeled with a fluorescent dye (YOYO-1).

Figure 2 shows the observations on the generation of micro w/w droplets in the PEG/DEX solution coexisting with (a) red blood cells (RBC), (b) epithelial cells (NMuMG cells), and (c) DNA (salmon sperm). The upper and lower panels in Fig. 2a show the w/w droplets generated along a glass capillary and on a planar confinement with a pair of horizontal glass plates (depth: ca. 100 μm), respectively. For both confinement with a glass capillary and between glass slides, RBC are mainly entrapped inside the droplets, as clearly revealed in the fluorescence microscopic images, where the cells are stained by Nile Red. The size of the droplets is rather uniform in the capillary experiments, in contrast to the size dispersion under confinement with glass slides. Figure 2b shows that the epithelial cells are localized on the interface of the w/w droplets, where fluorescence is emitted by mCherry expressed inside the cells. A similar localization of RBC and epithelial cells for w/w droplets in a PEG/DEX solution was observed under non-confinement conditions in the 3D experimental system as recently reported^33,34^. Figure 2c shows the generation of the droplet array entrapping DNA molecules, where DNAs are stained by YOYO-1. Here again, it is noted that DNA molecules are spontaneously concentrated inside the linearly-arranged droplets rich in DEX.

### Numerical modeling

Next, we performed a numerical simulation of the formation of micro w/w droplets under confinement to clarify the mechanism of the marked difference between the different geometries: in a glass capillary and between planar glass plates, which hereafter are called ‘tubule’ and ‘planar-gap’, respectively. For simplicity, we adopted two-dimensional modeling for tubule and planar-gap confinement. As in the above Experimental Results, we performed measurements in the aqueous solution with PEG and DEX under conditions that exhibit w/w phase-separation. Thus, just after mechanical mixing, for the standing solution we may expect a coarsening process of phase-separation, corresponding to spinodal decomposition. It may be reasonable to adopt the Cahn–Hilliard Equation^35–41^ to interpret the essence of the observed phenomena:

1 $$\frac{\partial \eta }{\partial t}= \nabla \left({M}_{c}\nabla \frac{\delta F}{\delta \eta }\right),$$

where the free energy $F$ has two different contributions: bimodality with the order parameter and the interfacial energy. Here, ${M}_{c}$ is a parameter of diffusivity and $t$ is time.

2 $$F= \int \left(RT[\eta \mathrm{ln}\eta +\left(1-\eta \right)\mathrm{ln}\left(1-\eta \right)]+L\eta \left(1-\eta \right)+\frac{\alpha }{2}{\left|\nabla \eta \right|}^{2}\right)dV,$$

where $L$, $\alpha$ and $dV$ are an interaction parameter, gradient energy coefficient and differential volume, respectively. The parameter, $\eta =[\mathrm{0,1}]$, represents the composition of PEG and DEX solution; $\eta =1$ corresponds to the state of 100% DEX without PEG. The first and second terms in the parentheses correspond to entropic contributions of mixing. The third term is the interaction energy, for which a parabolic relationship was adopted for simplicity and generality. We used the universal gas constant $R =8.31$ J/mol∙K and temperature $T =293$ K. For the calculation of Eq. (2), we tentatively adopted the parameters $L =7.0 \times {10}^{3}$ J/mol and $\alpha =6.0 \times {10}^{-7}$ Jm^2^/mol, so as to reproduce the experimentally observed process of segregation under a similar spatial scale and observation time.

**Figure 3:**
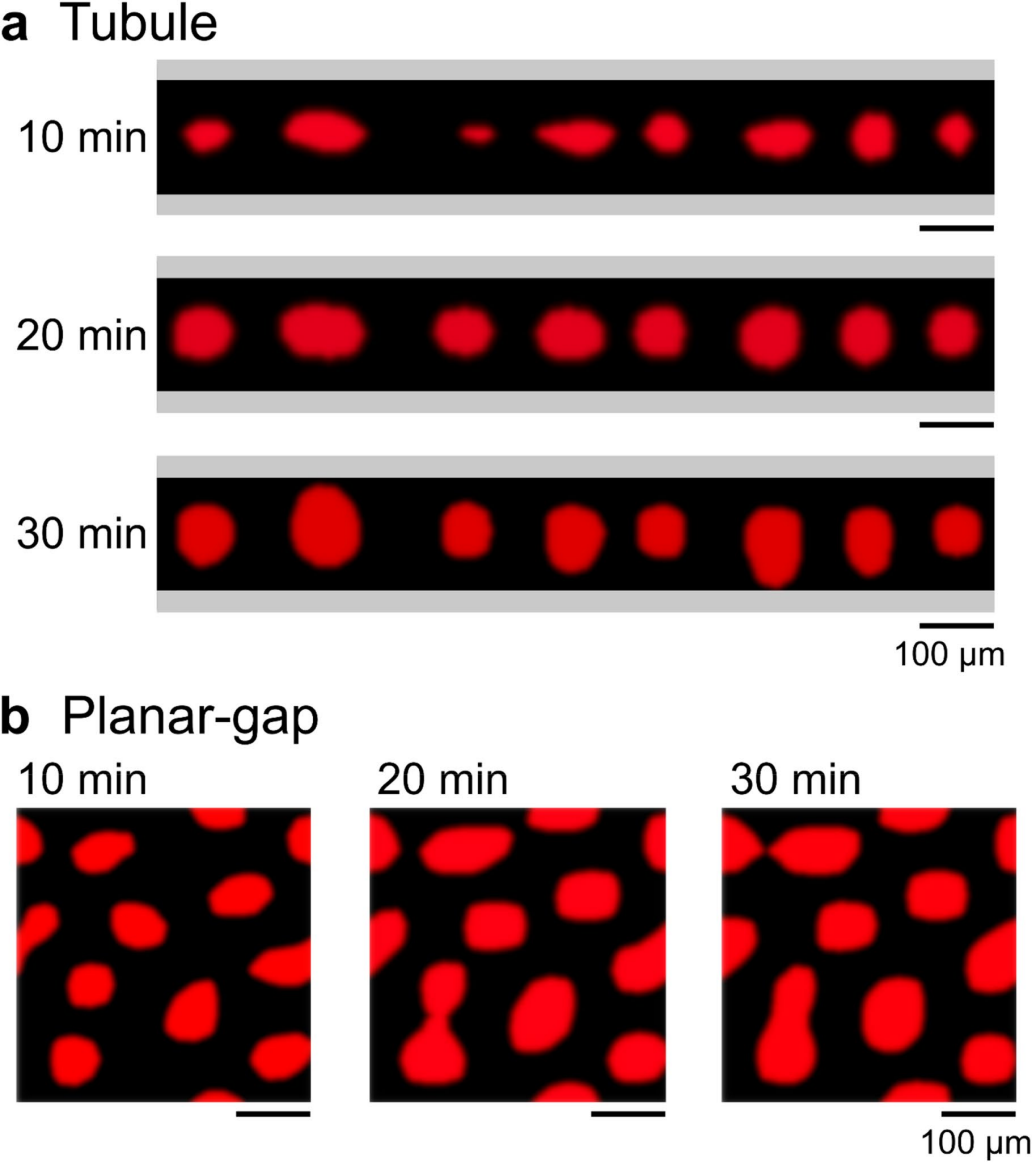
Numerical results regarding the time-development of micro-droplets. The numerical simulation for the phase-segregation was performed with the model equations (Eqs. (1) – (3)). The red region $(\eta \ge 0.6$) corresponds to DEX-rich droplets and the black region represents PEG-rich solution. (**a**) With Tubule confinement, the phase-segregated droplets with varied morphology tend to transform into a circular shape of similar size over time. The gray area represents the glass wall of the capillary. In the simulation, the boundary condition along the surface was taken to follow the conservation law and to prefer the PEG-rich solution $(\eta <0.5$). In contrast, for confinement with a Planar-gap as in (**b**), droplets tend to merge into larger droplets over time.

For the segregation between PEG and DEX as in Figs. 1 and 2, the PEG-rich solution and the DEX-rich droplets correspond to $\eta < 0.5$ and $\eta > 0.5$, respectively. Since the order parameter $\eta$ is dependent primarily on the relative composition of the PEG and DEX solution, we may need to consider that the diffusivity ${M}_{c}$ is sensitively dependent on $\eta .$ Here, we adopt a simple dependence with a bimodal relationship, $\eta \left(1-\eta \right)$;

3 $${M}_{c }= \left[\frac{{D}_{0}}{RT}\right]\eta \left(1-\eta \right),$$

where ${D}_{0}$ is the diffusion constant. In the simulation, we simply set ${D}_{0} =1.0 \times {10}^{-11}$ m^2^/s independent on the polymer composition, which value is two-order smaller than the self-diffusion of water. Regarding the boundary condition, for the planar-gap and both ends of the tubule, a periodic boundary condition was adopted. For the calculation for tubular confinement, as a model of the capillary experiments, we took into account the chemical effect of inner surface of the glass capillary. In the experimental observation as in Fig. 1, it is apparent that the surface region of the glass capillary is occupied with the PEG-rich solution and the DEX-rich droplet does not attach to the glass surface. This experimental trend indicates the difference in affinity between the segregated solutions; i.e., PEG has high affinity for the wall of the glass tube, while DEX has low affinity. Thus, we adopted the boundary condition that the solution facing the surface tends to become the PEG-rich condition, i.e., $\eta <0.5$. Figure 3 shows the time-dependent change in w/w segregation by using the time parameter, ${\Delta t}_{\mathrm{Num}}$. We carried out the simulation by adopting perfect homogeneity ($\eta = 0.4$) for the initial condition. On the other hand, in the actual experiments, we introduced the PEG/DEX aqueous solution into the glass capillary or into the planar confinement between glass plates with a parallel orientation, after mechanical mixing of the segregated solution. In other words, the initial condition in the experimental observation corresponds to the initial stage of spinodal decomposition, exhibiting heterogeneity that reflects the kinetic effect of phase-segregation (for a detailed condition, see "Numerical details"). Figure 3 exemplifies the micro w/w droplets that accompanied w/w phase segregation, corresponding to the tubule and planar-gap confinements, where coloring of the droplets is carried out with a threshold value of $\eta = 0.6$. Figure 3a shows that uniform-sized droplets are arranged along the long-axis of the tubule and tend to preserve their size without fusion between the droplets. In contrast, Fig. 3b shows the size dispersion of droplets corresponding to the planar-gap, and the droplets grow by fusion over time. Note that the manner of formation of micro w/w droplets varied markedly depending on the boundary condition. These results of a numerical simulation reproduce the essential behavior of the formation of micro w/w droplets in the PEG/DEX solution.

## Discussion

As shown in Figs. 1 and 2, cell-sized w/w droplets are generated inside the glass capillary in a self-organized manner, under the condition of phase segregation containing a binary polymer. In the usual 3D experiment without any confinement by the container of the solution, the size of the w/w droplets exhibits a rather broad distribution on the order of μm-mm as in Figs. S1 and S2. In contrast, here we have shown that, through the simple procedure to introduce the biopolymer solution into a glass capillary, uniform cell-sized droplets are generated in a self-organized manner. It may be of scientific value to extend the discovery of the present study so as to explore the suitable experimental conditions, such as composition of the solution, size and hydrophilicity of the narrow channel, to obtain stable, uniform droplets. In addition, it has been revealed that DNA molecules as well as living cells are spontaneously incorporated into the generated droplets, as has been observed for w/w droplets in bulk binary polymer solutions^33^.

The selective entrapment of DNA in DEX-rich droplets is attributable to the difference in the manner of assembly among polymers between PEG and DEX; i.e., a nanosized void space exists in the DEX-rich phase because of its stiff backbone and branched conformation, whereas the PEG-rich phase is fully occupied with flexible random coiled chains. For double-stranded DNA with a diameter of about 2 nm and a size of 500–1000 bp, the contour length is 1.5–3 μm. Thus, the DNA molecules can penetrate into the nanosized void space in DEX-rich droplets. On the other hand, because of the depletion interaction^27^ of PEG molecules as a flexible polymer on DNA as a stiff chain with large persistence length (ca. 50 nm), DNA molecules are repelled from the PEG-rich phase^42,43^. The difference in cell localization observed in the present study is attributable to various factors related to differences in cell properties, including their sizes, morphologies, surface structures, adhesive (non-adhesive) behaviors, number density, etc.

In conclusion, linearly-arranged cell-sized w/w droplets were spontaneously generated inside a glass capillary, where the generated droplets remained in a stationary state without coarsening. In contrast, the size of the droplets is varied and they tend to merge with each other over time under confinement in a planar-gap, as is well-known for the phenomenon of spinodal decomposition^44^. The emergence of uniform w/w droplets with such a simple methodology may be suitable for the construction of protocells or artificial cells. Since it has been found that w/w droplets are spontaneously covered by a phospholipid membrane^31^, in the near future it may become possible to construct a cell-like structure entrapping DNA and other macromolecules through the simple mixing of an aqueous solution.

## Methods

### Binary polymer solution to cause w/w micro-droplets

An aqueous two-phase system consisting of a chain-structured flexible polymer, polyethylene glycol (PEG) (Mw 7300–9300 Da, Fuji Film Wako Pure Chemical Industries, Osaka, Japan), and a branched stiff polymer, Dextran (DEX) (Mw 180,000–210,000 Da, Fuji Film Wako Pure Chemical Industries, Osaka, Japan) was adopted. Based on the results obtained in our recent systematic study, here we used the composition PEG:DEX = 5 wt%:5 wt%, which corresponds to a phase-separated state near the binodal line of the phase-separation, and micro w/w droplets of tens to hundreds μm rich in DEX emerged in the outer solution of rich in PEG^29,32^. For visualization of the DEX-rich phase with fluorescence microscopy, fluorescein isothiocyanate bound DEX (FITC-DEX, average Mw 250,000 Da, Ex: 488 nm, Em: 520 nm, Sigma-Aldrich, St. Louis, MO, USA) was used. For the experiment in the presence of living cells, 0.9% NaCl solution (Fuji Film Wako Pure Chemical Industries, Osaka, Japan) was used. For the other experiments, purified water (Milli-Q, 18.2 MΩ cm) was used.

### Experiments with a glass capillary

DNA or cells were added to a PEG/DEX binary solution and sucked up into a glass capillary (EM Meister Mini Caps(R), As One Corporation, Osaka, Japan) as in Fig. 1a, immediately after mechanical mixing by using a vortex mixer until the solution becomes cloudy, i.e. the micro-sized droplets (~ 100 μm) emerge. It was confirmed that degree of mixing strength does not induce apparent change in the size-distributions of droplets. The glass capillary has an inner diameter of 140 µm and a long-axis length of 32 mm. Both ends of the capillary were then blocked with a sealant to eliminate flow during microscopic observation. Without a sealant, slow directional flow was generated spontaneously as shown in Fig. S3. Where the linearly-arranged droplets moves accompanied by the gradual fusion of smaller droplets and, as the result, the generation of the almost equal-sized droplets.

### Cells

Two types of mammalian-derived cells were used: red blood cells and epithelial cells. For red blood cells (RBCs), the preserved blood of horse was purchased from Nippon Bio-test Laboratories Inc. (Saitama, Japan) and added 1% of the blood to PEG/DEX solution in saline solvent without any washing. For observation by fluorescent microscopy, Nile Red (Ex: 533 nm, Em: 637 nm, Fuji Film Wako Pure Chemical Industries, Osaka, Japan) was mixed with PEG/DEX solution at 40 µM to stain RBCs. As epithelial cells, we used NAMRU mouse mammary gland epithelial cells (NMuMG cells), which were cultivated from cultured cells at regular intervals^45^. NMuMG cells express the fluorescent ubiquitination-based cell cycle indicator (Fucci2) and exhibit fluorescence of mCherry (Ex: 515 nm, Em: 528 nm) in the Gap1 (G1) phase. We observed these cells when most of the cells were in the G1 phase. NMuMG cells were first separated from the culture medium by centrifugation and then transferred into the PEG/DEX solution.

### DNA

DNA from salmon sperm (500–1,000 bp, Fuji Film Wako Pure Chemical Industries, Osaka, Japan) was used. Crystalline powder was dissolved in nuclease-free water and added to PEG/DEX solution to a concentration of 150 µM. The antioxidant 2-mercaptoethanol (2ME, Fuji Film Wako Pure Chemical Industries, Osaka, Japan) was added at 4%. Tris(hydroxymethyl)aminomethane-HCl buffer (1 M Tris–HCl, pH 7.5, Nippon Gene, Tokyo, Japan) was added to the solution at 8 mM. The fluorescent cyanine dye YOYO-1 (Ex: 491 nm, Em: 509 nm, Molecular Probes, Inc., Eugene, OR, USA) was added at 0.2 µM to stain DNA.

### Microscopy measurements

Images were obtained with a fluorescent microscope. EVOS FL (Thermo Fisher Scientific, Waltham, MA, USA) was used for the observation of 4X images with a glass capillary. For more detailed observations, an Axio Observer. A1 (Carl Zeiss, Germany) equipped with a 40X objective lens was used and images were obtained with a CCD digital camera (C11440, Hamamatsu Photonics, Hamamatsu, Japan). All observations were conducted under ambient temperature (20–24 ℃).

### Numerical details

We carried out numerical calculations with a Cahn–Hilliard type equation by modifying the source code in Python available from the open access version^46^, provided by the Yamanaka Laboratory at Tokyo University of Agriculture and Technology, Japan. For simplicity, we performed numerical calculations with a two-dimensional system. For the calculation of tubular confinement, we adopted a boundary condition that the solution facing the surface tend to attract PEG and repel DEX, under no-flux boundary condition at the surface. In other words, around the boundary layer, the PEG-rich solution ($\eta < 0.5$) prefers the glass wall, while DEX-rich solution ($\eta > 0.5$) escapes from the wall, by keeping the mass-conservation condition. Since the volume of the PEG phase was greater than that of the DEX droplet in the actual experiment, we carried out the simulation by adopting the initial value ($\eta = 0.4$) on the PEG side, and perturbation from the initial value was given at random. The elapsed time in the experiment and in numerical calculations were represented by ${t}_{\mathrm{Exp}}$ and ${t}_{\mathrm{Num}}$, respectively. We carried out the simulation by adopting perfect homogeneity ($\eta = 0.4$) for the initial condition. However, the experimental observation corresponds to the initial stage of spinodal decomposition, not perfect homogeneity. In the actual experiment, phase-separation begins to occur at a nm-scale immediately after mechanical mixing. Thus, the timing of the beginning of observation for the tube in the experiment (${t}_{\mathrm{Exp}}= 0$ s) corresponds to ${t}_{\mathrm{Num}}= 400$ s in the simulation. We ran the calculation without the boundary condition from perfectly homogeneity from 0 s until 400 s, and then performed the simulation by introducing the boundary condition. Figure 3 shows the results of the time development by taking ${\Delta t}_{\mathrm{Num}}= 0$ s as the time after 10,000 steps (corresponding to 400 s) from the homogenous initial state. These results reveal the time development of micro w/w droplets in the PEG/DEX solution generated after ${\Delta t}_{\mathrm{Num}}$ = 10 min, 20 min and 30 min under the tubule and planar-gap in the simulation. The time step is 0.1 s, and step numbers are 10,000, 16,000 and 22,000, corresponding to time-periods of 1000 s, 1600 s and 2200 s. The grid spacing in the computation is taken as $2.0 \times {10}^{-5}$ m. The inner diameter of the tubule is 140 μm, which is the same as in the actual experiment.

## Supplementary Information

Supplementary Information.

## Data Availability

The data analyzed in this paper and computer code for numerical simulation are available from the corresponding author on the reasonable request.
