# Supplementary material for: Emergence of uniform linearly-arranged micro-droplets entrapping DNA and living cells through water/water phase-separation
Source: Sci Rep. 2021 Dec 7;11:23570. doi: 10.1038/s41598-021-02990-w (PMC8651656; doi:10.1038/s41598-021-02990-w)
Supplement: Supplementary file 1 — Supplementary Information. [file 41598_2021_2990_MOESM1_ESM.pdf]

Supplementary information for

Emergence of uniform linearly-arranged micro-droplets entrapping DNA and living cells through water/water phase-separation

Mayu Shono,[2,5] Ritsuki Ito,[1,5] Fumika Fujita,[1] Hiroki Sakuta,[*1,3] and Kenichi Yoshikawa[1,4]

[1] Faculty of Life and Medical Sciences, Doshisha University, Kyoto 610-0394, Japan

[2] Department of Chemical Engineering and Materials Science, Doshisha University, Kyoto 610-0321, Japan

[3] Organization for Research Initiatives and Development, Doshisha University, Kyoto 610-0394, Japan

[4] Center for Integrative Medicine and Physics, Institute for Advanced Study, Kyoto University, Kyoto 606-8501, Japan

[5] Contributed equally

[*] Corresponding Author (hsakuta@mail.doshisha.ac.jp, +81-774-65-7760)

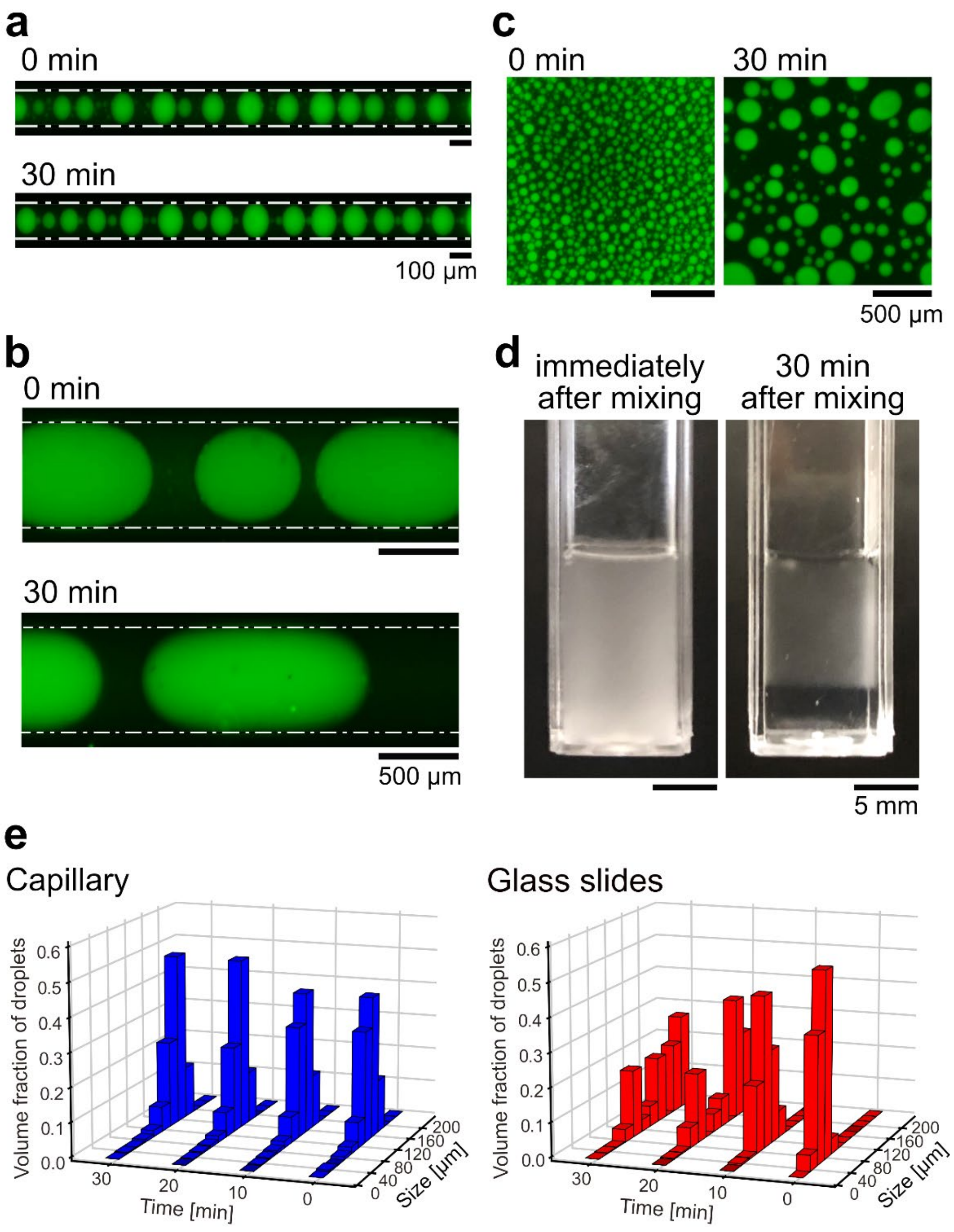

Figure S1 Comparison of the nature of w/w phase-separation with time, depending on the confinement conditions; (**a**) capillary with the diameter of 140 μm, (**b**) capillary with the diameter of 630 μm, (**c**) between glass slides, and (**d**) cm-sized cell. The solution composition is PEG:DEX = 5 wt%:5 wt%. For each confinement, the time-dependent change between the initial stage (0 min) after mechanical mixing and the late stage (30 min) is shown. Dashed lines indicate the inner glass wall. In (**a**), (**b**) and (**c**), the green region corresponds to DEX-rich solution. For the solution at 30 min after mixing in (**d**), the upper phase is PEG-rich, and the lower phase is DEX-rich, indicating macroscopic phase-separation. (**e**) Time development of the size distribution of droplets as revealed by the volume fraction, of which integral is normalized to be unity. In capillary, the droplets form narrower size distribution with time. Whereas in glass slide, distribution tends to be wider with time.

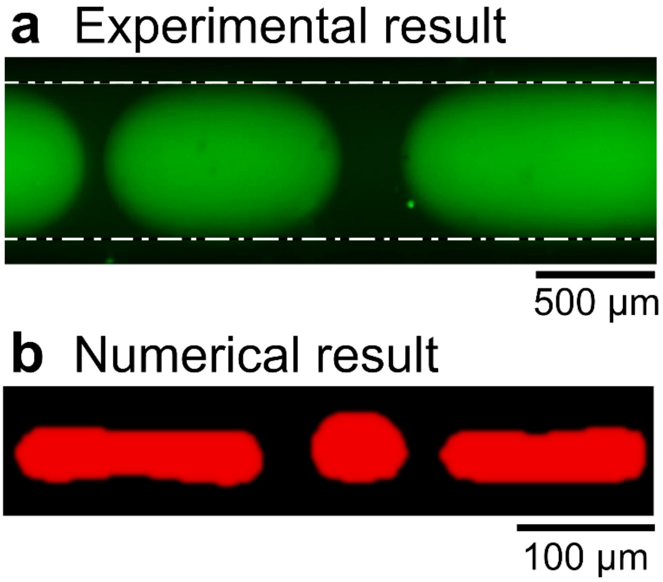

Figure S2 Non-spherical droplets were generated through the fusion of the neighboring spherical droplets when we used the larger size of glass capillary in the experimental result, as well as in the numerical simulation.

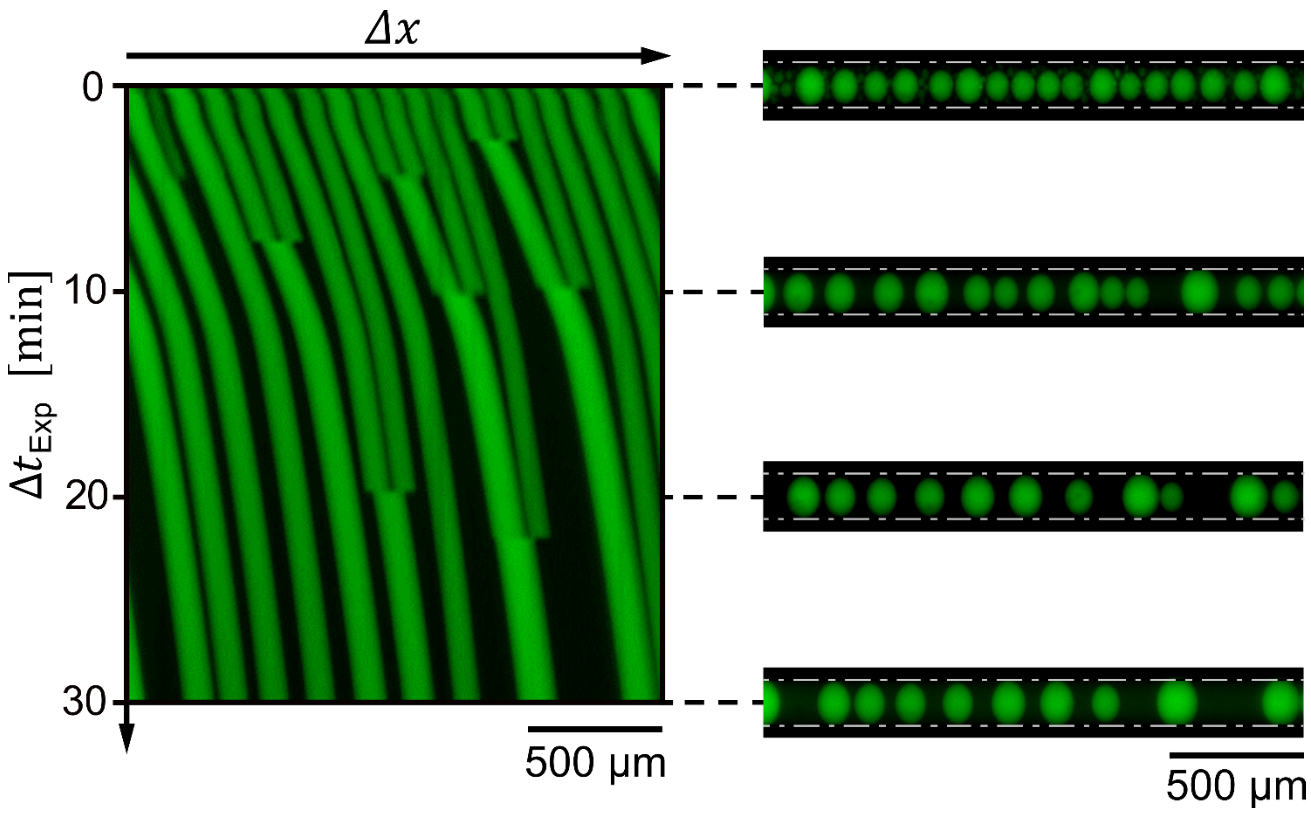

Figure S3 Spatio-temporal diagram to show the time-dependent change in phase segregation along the glass capillary in the presence of solution flow. The solution composition is the same as in Fig. 1**b**; PEG:DEX = 5 wt%:5 wt%. The panels on the right show snapshots of the capillary at different times. Time indicates the period after the start of the microscopic observation, which is ca. 2 min after the timing of mechanical mixing. Dashed lines indicate the inner glass wall. DEX was labeled with fluorescein isocyanate (FITC-DEX, Mw = 250,000).
